# Assessing the Kynurenine–Tryptophan Ratio (KTR) and CYP1 Activity in Longnose (*Catostomus catostomus*) and White Suckers (*Catostomus commersonii*) Exposed to Petroleum-Derived Contaminants from the Alberta Oil Sands Region

**DOI:** 10.3390/toxics13100862

**Published:** 2025-10-11

**Authors:** Laiba Jamshed, Amrita Debnath, Amica Marie-Lucas, Thane Tomy, Gregg T. Tomy, Tim J. Arciszewski, Mark E. McMaster, Alison C. Holloway

**Affiliations:** 1 Department of Obstetrics and Gynecology, McMaster University, Hamilton, ON L8S 4L8, Canada; jamshel@mcmaster.ca (L.J.);; 2 Centre for Oil and Gas Research and Development, University of Manitoba, Winnipeg, MB R3T 2N2, Canada; 3 Alberta Environment and Parks, Calgary, AB T2E 7L7, Canada; 4 Environment and Climate Change Canada, Burlington, ON L7S 1A1, Canada; mark.mcmaster@ec.gc.ca

**Keywords:** tryptophan metabolism, polycyclic aromatic compound, biomonitoring, environmental stress, biomarkers, species-specific responses, freshwater fish, enzyme activity, biometric indicators, CYPs

## Abstract

In the Alberta Oil Sands Region (AOSR), environmental stressors linked to oil sands industrial activity may have significant and species-specific impacts on local wildlife. This study evaluated the kynurenine–tryptophan ratio (KTR) as a potential biomarker for environmental exposure in longnose suckers (*Catostomus catostomus*) and white suckers (*Catostomus commersonii*) collected from various locations within the AOSR. The relationship between KTR and CYP1 enzyme activity (ethoxyresorufin-O-deethylase; EROD) was assessed alongside biometric indices, including gonadosomatic index (GSI), hepatic somatic index (HSI), and fat content. Both species exhibited increased EROD activity when exposed to oil sands natural deposits and potential industrial activity, indicating significant polycyclic aromatic compound (PAC) exposure. However, KTR changes were species-dependent: longnose suckers showed an inversely proportional relationship between KTR and EROD, while white suckers displayed a directly proportional correlation. Longnose suckers downstream of both municipal waste and industrial activity exhibited significant increases in GSI and fat content, with KTR varying more consistently by location rather than sex, suggesting that KTR may be a more reliable marker for location-based exposure. Species-specific differences in KTR and EROD relationships may be influenced by the distinct environmental requirements of each species, and their differing sensitivities to environmental conditions, including temperature, turbidity and flow conditions, during sampling periods. These findings illustrate the complexity of interpreting environmental biomarkers in wildlife and emphasize the need to consider ecological requirements and environmental conditions. Further research is necessary to validate this biomarker across different years and conditions and enhance its application in environmental monitoring and conservation efforts.

## 1. Introduction

In Canada, environmental monitoring is a key component of the Fisheries Act as a condition to authorize effluent deposition [1]. Monitoring of fish in wildlife is conducted by measuring contaminant levels, reproductive success indicators (e.g., gonad weight, fecundity) and overall health (e.g., liver weight, condition factor, age) in native species as indicators of ecosystem and individual health.

In the past decade, the Alberta Oil Sands Region (AOSR) has garnered attention for the potential cumulative ecological effects of oil sands operations on local wildlife and the aquatic environment. In partnership with the Government of Alberta and local Indigenous stakeholders, Environment and Climate Change Canada (ECCC) has worked to guide oil sands development towards environmentally responsible practices [2]. Among the organisms at risk, freshwater fish and aquatic species are particularly susceptible to chemical inputs introduced into watersheds by oil sands development [3]. Environmental monitoring for these vulnerable aquatic populations is crucial to determine the degree of contamination and its downstream effects on the local ecosystem.

The longnose sucker (*Catostomus catostomus*) and the white sucker (*Catostomus commersonii*) are native fish species commonly used for environmental monitoring in Canada due to their widespread abundance, robustness, and adaptability [4,5]. In environmental monitoring programs using fish, common biometric tools to assess energy metabolism and reproductive condition are the hepatic somatic index (HSI), the gonadal somatic index (GSI), and fat content [6,7]. While these measures provide valuable insights into aspects of fish health, they may not capture the full range of physiological responses to environmental stressors [2,8]. This limitation prompted us to explore more integrative markers of health. Tryptophan metabolism, which is involved in energy regulation, protein excretion, and serotonin production, plays a crucial role in growth, reproduction, and survival, suggesting that tryptophan or its kynurenine pathway metabolites could serve as a more comprehensive biomarker of overall health in fish [9].

Recent research has highlighted the kynurenine–tryptophan ratio (KTR) as a potential cross-species indicator of organism health [10,11]. KTR measures the relative proportion of key metabolites, kynurenine and tryptophan, that are involved in generating and managing stress and immunological responses in mammals, fish, and birds. Additionally, alterations in KTR have been implicated in responses to stress and environmental pollutants [10,12,13,14,15,16,17]. Among the few molecular markers of exposure that capture pathway-specific responses, cytochrome P450 enzymes are widely used in aquatic toxicology, with ethoxyresorufin-O-deethylase (EROD) activity serving as a the most common proxy for exposure to polycyclic aromatic compounds (PACs). More recently, evidence has emerged that tryptophan metabolism is also altered following exposure to PACs, suggesting it may provide complementary insight into organismal stress responses [18,19,20]. Given that the oil sands region is being heavily monitored and various biometric data is being collected in ongoing monitoring efforts, we hypothesized that the KTR may provide additional information as an integrated marker of organismal health. Therefore, this study aimed to assess the effects of the oil sands deposit and industrial activity in longnose suckers and white suckers from the oil sands region using metabolite levels, enzyme activity, and established biometric indices.

## 2. Materials and Methods

### 2.1. Fish Collection and Tissue Processing

Suckers (*Catostomus*) species are broadly distributed across North America, including established populations within Alberta’s Athabasca River watershed [21]. The fish sampling for this study was conducted as described in McMaster et al. (2018) [6] and Arciszewski and McMaster (2021) [22]. Briefly, white suckers and longnose suckers were collected in the fall (September–October) of 2013 and 2014. White and longnose suckers were collected across the river system at distinct locations: two reference sites outside of the oil sands deposit (Athabasca and Poacher’s Landing, collectively termed Reference): a deposit site upstream of development but downstream of the Fort McMurray municipal wastewater discharge (Northland, termed Deposit), and two or three sites located within both the deposit and oil sands development areas (Suncor, Muskeg, and Ells). For this study, adult female and male longnose suckers were collected in 2013 using boat electrofishing. Collections were conducted under scientific collection permits (13-0445, 14-0456) issued by Alberta Environment and Parks, and in accordance with Animal Use protocols (1315, 1415) approved by the Canadian Council on Animal Care through the National Water Research Institute Animal Care Committee. The white suckers assessed in this study were collected in 2014. Sample numbers per site and sex are provided in Supplemental Table S1. For all fish, after electroshock, fish were captured with dip nets (0.5 cm mesh size), placed in a livewell, and transported to the on-site mobile laboratory for processing. In the lab, fork length (FL; ±1 mm), body weight (BW; ±0.1 g), liver weight (LW; ±0.01 g), gonad weight (GW; ±0.01 g), and sex were recorded. Opercula were removed for age determination (±1 y). Portions of the livers were snap-frozen in liquid nitrogen and stored at −80 °C until analysis. Hepatic ethoxyresorufin-O-deethylase (EROD) activity, a marker of mixed-function oxygenase (MFO) activity was measured shortly after collection, with livers frozen at −80 °C during field sampling and subsequently analyzed within approximately six months of collection following Van den Heuvel et al. [23]. The remainder of each liver sample was maintained at −80 °C without freeze–thaw until later metabolite analysis, ensuring no repeated thawing of tissues. Visceral fat content was assessed using a qualitative fat index (1 = very low fat; up to 5 = very high fat) adapted from Munkittrick and Dixon (1988) [24].

### 2.2. Kynurenine–Tryptophan Ratio (KTR) Assessment

#### 2.2.1. Chemicals and Standards

Unlabeled analytical standards of L-tryptophan (TRP, ≥98% HPLC) and L-kynurenine (KYN, ≥98%) were obtained from Sigma- Aldrich Canada (Oakville, ON, CA); mass labeled L-tryptophan-2′,4′,5′,6′,7′-d5 (indole-d5) (98%, TRP-d5), L-kynurenine-d4 [4-(2-aminophenyl-3,5-d2)] (99%, KYN-d4) were sourced from CDN Isotopes (Pointe-Claire, QB, CA); and all solutions were stored at 4 °C. High-performance liquid chromatography (HPLC)-grade L-Acetonitrile (ACN) and water were purchased from Fisher-Scientific, while Ultrapure Milli-Q water was obtained through a Synergy™ Milli-Q purification system (Millipore, Billerica, MA, USA), designed to produce water of 18.2 MΩ·cm resistivity at 25 °C.

#### 2.2.2. Sample Preparation and Extraction

Approximately 0.02 g of fish liver was homogenized using a beadmill homogenizer (Precellys Evolution Touch, Bertin Technologies, Rockland, MD, USA) and subsequently transferred into a microcentrifuge tube and spiked with 10 μL of mass-labeled internal standards (TRP-d5 and KYN-d4, 25 ng/μL each). Samples were extracted by adding 1.35 mL of 35:65 H_2_O:ACN, followed by sonication in an ice bath for 12 min, vortexing for 1 min, and centrifugation at 14,800 rpm for 15 min at room temperature. The supernatant was removed, and the remaining tissue was re-extracted as before. Extracts were combined and reduced in volume to 1.5 mL with a gentle stream of ultra-high purity N_2_ prior to high-performance liquid chromatography tandem mass spectrometry (HPLC/MS/MS) analysis. Liver aliquots used for KTR measurement had been snap-frozen in liquid nitrogen at the time of collection, stored continuously at −80 °C, and were never subjected to freeze–thaw prior to extraction, where they were protected from heat, light, and oxygen; factors known to influence tryptophan stability [25,26].

#### 2.2.3. High Performance Liquid Chromatography Tandem Mass Spectrometry

TRP and KYN levels in fish tissue were detected and quantified using a previously validated protocol [12]. Separations of native and mass-labeled analytes were carried out on a SEQUANT ZICR HILIC PEEK coated HPLC column (4.6 mm × 100 mm, 3.5 μM particle size, 200 A pore size) (Merck, Darnstadt, Germany). The HPLC system was coupled to a Sciex 365 triple quadrupole mass spectrometer retrofitted with an HSID Ionics EP+ orthogonal ionization source. Positive ion mode was used for ionization, with multiple reaction monitoring ion transitions used for detection and quantitation as follows: TRP: *m*/*z* 205.1 → 188.2; KYN: *m*/*z* 209.4 → 94.1, TRP-d5: *m*/*z* 201.2 → 192.4 and KYN-d4: *m*/*z* 213.3 → 196.2. Method detection limits, calculated using the Eurachem Guide for Method Validation [27] were 133.9 ng/g for KYN and 89.8 ng/g for TRP.

### 2.3. Data and Statistical Analysis

Statistical analyses were performed in GraphPad Prism (v.9.5.1, GraphPad Software, San Diego, CA, USA). Outliers were identified with Grubb’s Test, normality was assessed using the Shapiro–Wilk test and data were checked for equal variance. When comparing reference sites against other locations, one-way analysis of variance (ANOVA) was performed, with Dunnett’s post hoc test applied for pairwise comparisons where significant differences were detected (*p* ≤ 0.05). Non-normally distributed data or those with unequal variance were analyzed with Kruskal–Wallis one-way ANOVA on ranks, followed by Dunn’s post hoc test when significant differences were found. The data are reported as mean ± standard error of mean (SEM), with significance defined as *p* ≤ 0.05.

To investigate relationships between variables, Pearson’s correlation analysis was performed to compare EROD activity and KTR levels using simple linear regression. Further profiling included Pearson’s correlations between EROD activity, KTR levels, weight, condition factor, HSI, GSI, and fat content for each fish type.

Additionally, variation based on sex and location was analyzed using two-way ANOVA to assess the influence of these factors on outcome measures. Interactions between sex, location, and physiological parameters were also examined to determine site-specific and sex-specific responses.

## 3. Results and Discussion

### 3.1. Comparative Analysis of Kynurenine–Tryptophan Ratio and EROD Activity in Longnose and White Sucker

In field studies, fish exposures serve as critical tools for monitoring the quality of aquatic environments. In eco-epidemiology, fish are an important model organism, widely used in pollution monitoring programs [28]. Typical assessments focus on screening for visible signs of disease and abnormalities, and biochemical markers, including tissue contaminant burdens and metabolic stress indicators (e.g., lipid content and condition factor), to provide insights on the health of fish populations and their environments [29]. Biomonitoring of fish health is of considerable importance, particularly in regions where there is potential for industrial activities to affect aquatic environments.

**Figure 1:**
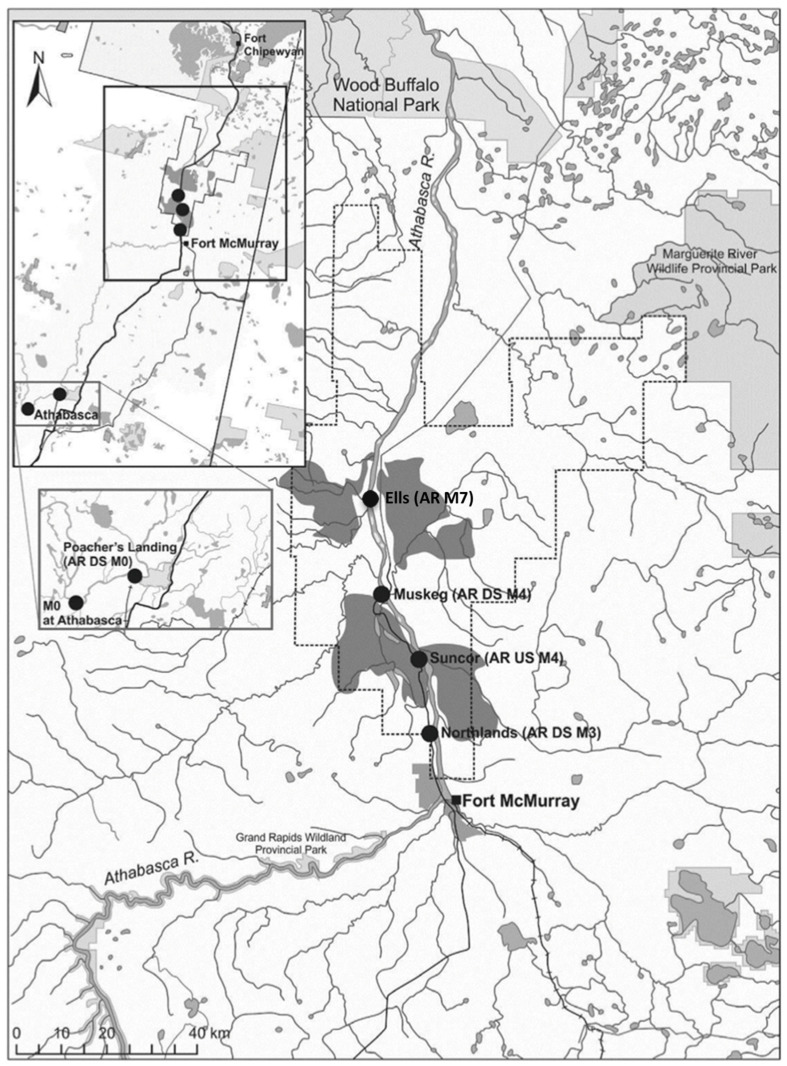
Sampling locations used to assess the impact of oil sands activity on the kynurenine:tryptophan ratio (KTR) in fish. Fish were collected from five or six sites along the Athabasca River system: two reference sites outside of the oil sands formation (Athabasca River and Poacher’s Landing), one site within the deposit zone (Northland), and two to three sites downstream of oil sands industrial activity (Suncor, Muskeg, and Ells). These locations were selected to capture potential gradients of exposure related to industrial development.

The Catostomidae family includes more than 76 freshwater species that occupy a wide range of habitats across North American ecosystems. These fish face numerous challenges, including migration barriers, variations in water levels and temperatures, environmental contamination, habitat degradation, resource exploitation, and the effects of non-native species [30]. In Alberta, *Catostomus* species are particularly affected by temperature fluctuations and environmental pollutants, making them valuable indicator species in monitoring natural variability and anthropogenic stressors within the Athabasca watershed [6,30,31]. To evaluate the influence of oil sands operations on KTR, fish were collected from five or six sites within the Athabasca River system, including reference sites outside of the oil sands formation (Athabasca River and Poacher’s Landing), a site within the oil sands deposit (Northland), and two or three sites downstream of oil sands industry activity (Suncor, Muskeg, Ells) (Figure 1). 

**Figure 2:**
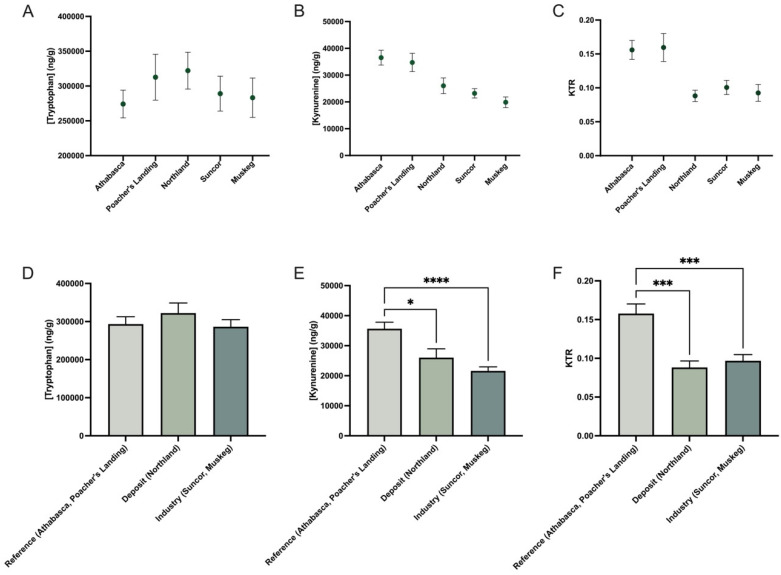
Kynurenine–tryptophan ratio (KTR) of longnose sucker (*Catostomus catostomus*) across five collection sites (**A**–**C**), pooled male and females (~40), and then grouped according to reference (Athabasca, Poacher’s Landing), oil sands deposits and sewage (Northland), and oil sands deposit and industry activity (Suncor, Muskeg) (**D**–**F**). The data are presented as mean ± SEM. Statistical significance compared to control is denoted by asterisks: * *p* < 0.05, *** *p* < 0.005, **** *p* < 0.001.

**Figure 3:**
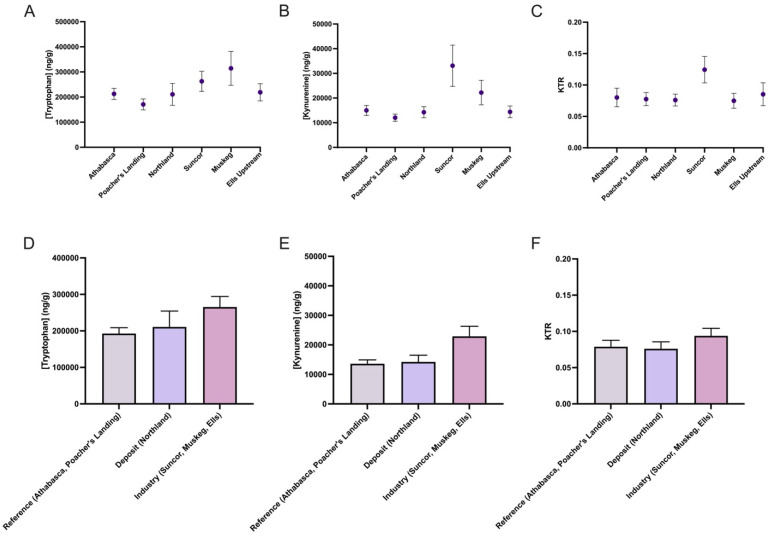
Kynurenine–tryptophan ratio (KTR) of white sucker (*Catostomus commersonii*) across five collection sites (**A**–**C**), pooled male and females (~10), and then grouped according to reference (Athabasca, Poacher’s Landing), oil sands deposits and sewage (Northland), and oil sands deposit and industry activity (Suncor, Muskeg, Ells) (**D**–**F**). The data are presented as mean ± SEM.

The longnose sucker (*Catostomus catostomus*) was assessed across five collection sites and data were pooled from both sexes (Figure 2). Significant differences in KTR were observed between reference sites and both deposit and sites near industry (Figure 2C), indicating location-specific alterations in the kynurenine pathway. This decrease in KTR was primarily driven by reduced kynurenine levels, while tryptophan levels remained unchanged. In contrast, the white sucker (*Catostomus commersonii*) did not show statistically significant changes in KTR across collection sites (Figure 3). However, there was a noticeable trend of increased kynurenine levels at Suncor and Muskeg but not Ells, though these differences did not reach statistical significance.

Fish react to environmental stress through two primary hormonal axes: the sympatho-chromaffin (SC) axis and the hypothalamic-pituitary-interrenal (HPI) axis [32]. Cortisol is a main hormonal mediator of the stress response in fish. Importantly, stress has been shown to alter the KTR in fish [12]. Our group previously found that rainbow trout (*Oncorhynchus mykiss*) subjected to acute stress exhibited increased KTR in liver and brain tissues 48 h post-exposure, which correlated with elevated cortisol levels [12]. Although we have not measured cortisol levels in this study, this prior evidence suggests a potential link between endocrine stress responses and KTR variation that could be explored in future studies of wild-caught fish.

The observed decrease in KTR in longnose but not white suckers may reflect the differing ecological requirements and sensitivities of the two species. Longnose suckers are known to require more specialized environments, often favoring cold, clear waters and specific latitudinal ranges for stable environments with minimal turbidity [33,34,35]. Changes to their environment, such as increased temperature, pollution, or altered flow conditions, can disrupt the longnose sucker’s stress response, potentially overwhelming their ability to maintain homeostasis and leading to shifts in metabolic pathways like the tryptophan-kynurenine pathway. In contrast, white suckers did not show significant changes in KTR. Studies have shown that white suckers can tolerate a range of environmental conditions, including variations in nutrient loading [6,36]. Indeed, Quinn et al. (2010) [31], who examined Mountain Whitefish (*Prosopium williamsoni*) and White Sucker exposed to temperature and agrochemicals in Alberta’s Oldman River, found species-specific differences in acetylcholine esterase activity and physiological stress responses, suggesting that white suckers were more resistant to temperature and pesticide stress. This resilience may explain the absence of a measurable KTR response to industrial inputs in the different sampling regions. However, without cortisol levels to test this hypothesis, this remains speculative. Additionally, differences in sample size and collection year must also be considered. Longnose suckers were collected in 2013, with a robust sample size of 20 males and 20 females per site, while white suckers were collected in 2014, a year characterized by unusually low river flow and the warmest temperatures of the six years of available data, with only 10 total fish sampled per site [22]. This combination of low flow and high temperatures could have affected water quality and bitumen-derived contaminant distribution, potentially influencing the physiological responses observed in the white suckers [37]. This difference in sampling year and sample size introduces variability that complicates direct comparisons of exposures between the two species, particularly for endpoints measured in 2014. Further investigations with larger sample sizes collected at the same time would need to confirm that the observed site-specific changes in KTR are species-dependent. Notably, the variability in KTR was lower in white suckers compared to longnose suckers, which suggests a more stable response despite the smaller sample size.

An alternative explanation for differences in KTR responses between species and locations may be related to the impact of bitumen-related compounds on aryl hydrocarbon receptor (AhR)-signaling pathways. AhR is activated by various environmental pollutants, including polycyclic aromatic hydrocarbons (PAHs), which are abundant in the AOSR and industry effluent [38,39]. Activation of AhR leads to downstream metabolic changes, including the induction of cytochrome P450 enzymes, particularly CYP1A. Xenobiotics and endogenous compounds, which act as AhR ligands, increase the expression of CYP1, which can be monitored by assessing ethoxyresorufin-O-deethylase (EROD) activity, a common biomarker of AhR activation and CYP1A activity. Numerous laboratory and field studies have established EROD as a reliable indicator of exposure to industrial effluents, contaminated sediments, chemical spills, and PACs (Reviewed In: [40,41]).

**Figure 4:**
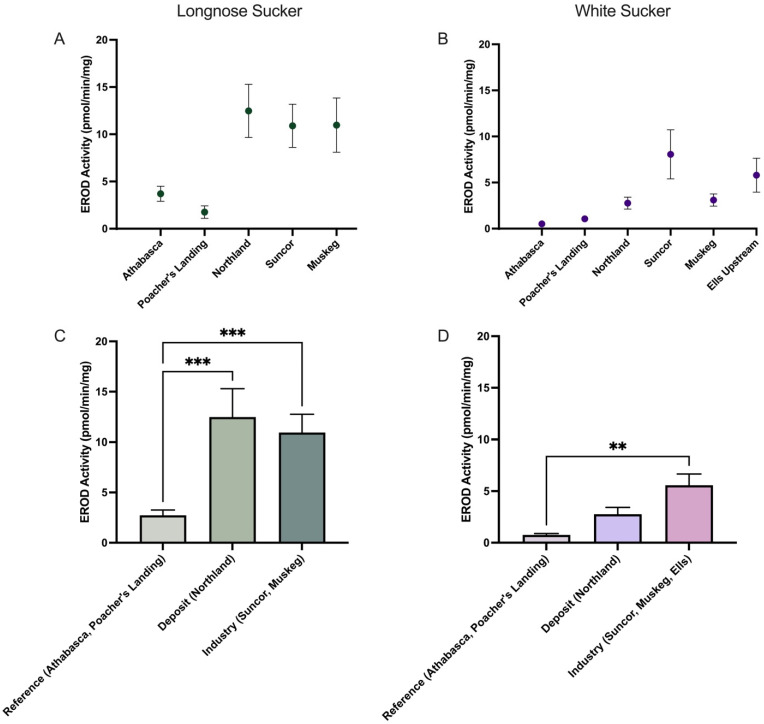
EROD activity (pmol/min/mg) of Longnose Sucker (*Catostomus catostomus*) (**A**,**C**) pooled male and females (~40), and White Sucker (*Catostomus commersonii*) (**B**,**D**) pooled male and females (~10), across 5 or 6 collection sites and grouped according to reference (Athabasca, Poacher’s Landing), oil sands deposits and sewage (Northland), and oil sands deposit and industry activity (Suncor, Muskeg, Ells). The data are presented as mean ± SEM. Statistical significance compared to control is denoted by asterisks: ** *p* < 0.01, *** *p* < 0.005.

**Figure 5:**
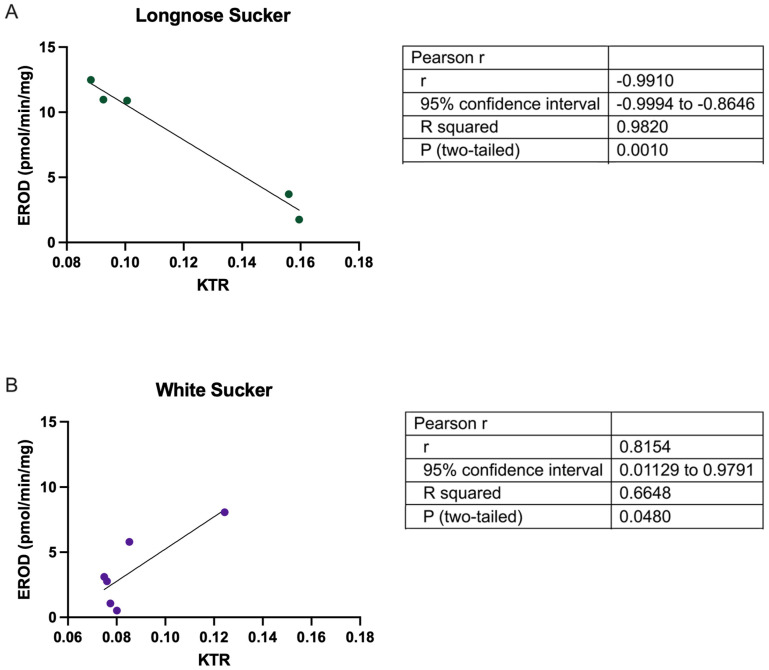
Pearson’s correlation between EROD activity and KTR levels in (**A**) longnose sucker (*Catostomus catostomus*) and (**B**) white sucker (*Catostomus commersonii*). Values represent site-level means ± SEM across five locations (longnose sucker) and six locations (white sucker). Regression lines indicate linear fits across site means; EROD activity is plotted on the y-axis and KTR on the x-axis.

In this study, EROD activity was significantly elevated in longnose sucker from both the deposit and industry sites compared to reference locations (Figure 4A,C). This increase in EROD activity suggests a heightened level of AhR activation and CYP1A induction in longnose sucker in response to bitumen exposure, regardless of the source. In white sucker, EROD activity was significantly higher only at the industry sites, with no significant change observed at the deposit site (Figure 4B,D). Interestingly, AhR ligands have also been demonstrated to increase tryptophan catabolism leading to increased levels of kynurenine metabolites, which are also AhR ligands. Indeed, it is well-established that derivatives of tryptophan metabolism, particularly those that are photo-oxidized, can bind to AhR with high affinity and serve as ideal substrates for CYP1 enzymes, suggesting that tryptophan metabolism can influence AhR activity [42,43]. In this study, a statistically significant negative correlation was observed between EROD activity and KTR levels in longnose suckers (r = −0.9910, *p* < 0.0010) (Figure 5), whereas in white suckers, a statistically significant positive correlation between EROD activity and KTR levels was found (r = 0.8154, *p* = 0.048). These differences suggest species-specific responses to environmental exposures, potentially reflecting differences in baseline stress tolerance, AhR sensitivity or downstream signaling, and regulation of tryptophan-catabolizing enzymes such as indoleamine 2,3-dioxygenase and tryptophan 2,3-dioxygenase [31]. While KTR is mechanistically linked to immune activation, inflammation, and broader metabolic stress, its relationship to organismal health and whole-body condition remains under investigation. To explore whether KTR reflects more systemic biological effects, we examined its association with biometric indicators including body weight, condition, liver and gonad indices (HSI, GSI), fat content, and sex.

### 3.2. Does KTR Correlate with Biometric Data?

**Figure 6:**
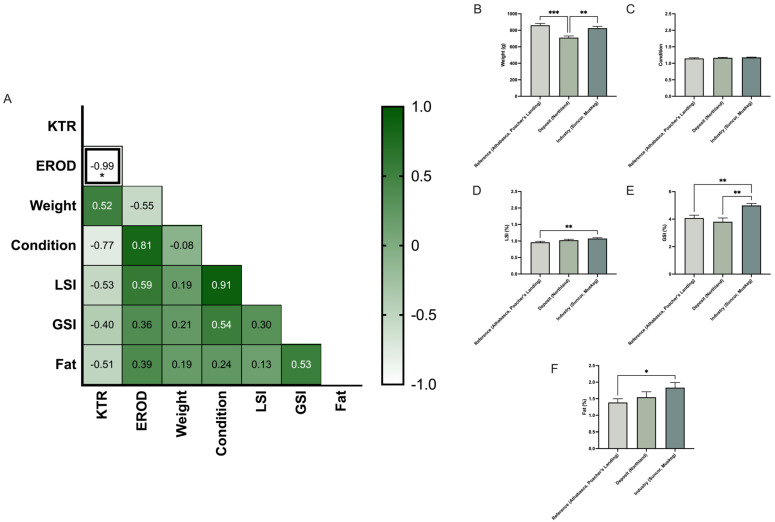
Profiling the longnose sucker (*Catostomus catostomus*). (**A**) Pearson’s correlation matrix analysis between ethoxyresorufin-O-deethylase (EROD) activity, kynurenine–tryptophan ratio (KTR), weight, condition, hepatic somatic index (HSI), gonadosomatic index (GSI), and fat. The heatmap color scale represents correlation coefficients (r), ranging from −1 (light green) to +1 (dark green). (**B**–**F**) Bar graphs of biometric indices: (**B**) weight, (**C**) condition, (**D**) Hepatic somatic index, (**E**) Gonado Somatic Index, and (**F**) fat content, measured in pooled males and females (~40 fish total) across five collection sites. Sites were grouped according to reference (Athabasca, Poacher’s Landing), oil sands deposits and sewage (Northland), and oil sands deposit and industry activity (Suncor, Muskeg). The data are presented as mean ± SEM. Statistical significance is denoted by asterisks: * *p* < 0.05, ** *p* < 0.01, *** *p* < 0.001.

**Figure 7:**
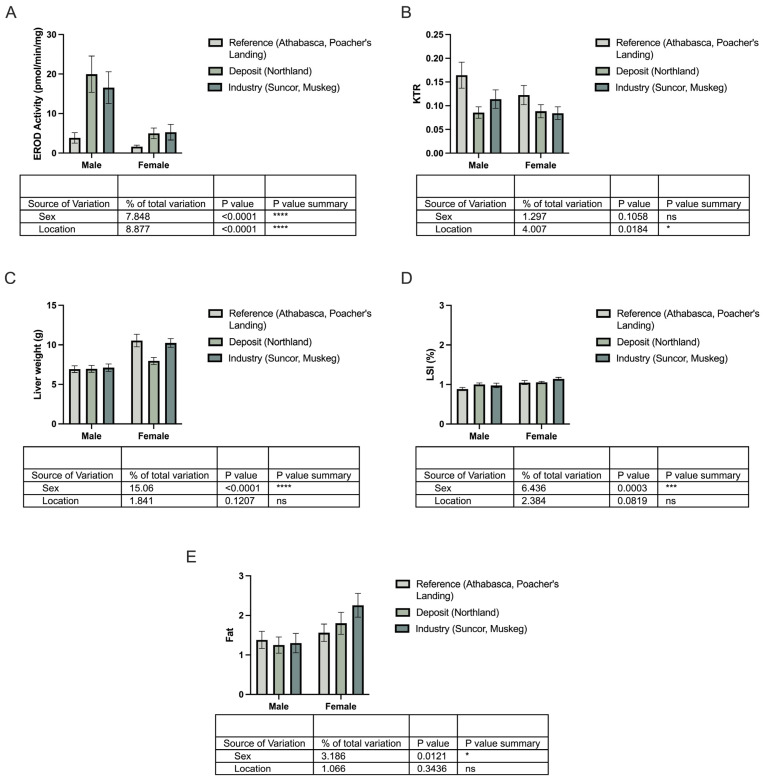
Profiling the longnose sucker (*Catostomus catostomus*) by sex and location. Two-way ANOVA was used to evaluate the effects of sex and location on (**A**) ethoxyresorufin-O-deethylase (EROD) activity, (**B**) kynurenine–tryptophan ratio (KTR), (**C**) liver weight, (**D**) hepatic somatic index (HSI), and (**E**) fat content. Bars represent mean ± SEM for males and females, with three bars per sex corresponding to reference sites (Athabasca, Poacher’s Landing), oil sands deposits and sewage (Northland), and oil sands deposit and industry activity (Suncor, Muskeg). Statistical significance relative to the reference group is denoted by asterisks: * *p* < 0.05, *** *p* < 0.005, **** *p* < 0.001.

In longnose sucker, correlation analysis revealed that KTR and EROD activity were the only variables significantly correlated (Figure 6A). While EROD activity varied significantly by both sex and location, KTR was influenced only by location, indicating that environmental contaminants are the dominant factor driving changes in tryptophan metabolism in this species (Figure 7). HSI, GSI, and fat were significantly different between reference and industry sites, but not between reference and deposit sites (Figure 6D–F). The marked reduction in GSI, which reflects the energy invested in gonadal development, at industry sites compared to both reference and deposit sites suggests that reproductive health is being adversely impacted by environmental contaminants specifically associated with industry activity. Although increased tryptophan catabolism to kynurenine has the potential to reduce the amount of tryptophan used as substrate for serotonin, an important mediator of fish reproduction [44], there was no relationship between KTR and GSI.

**Figure 8:**
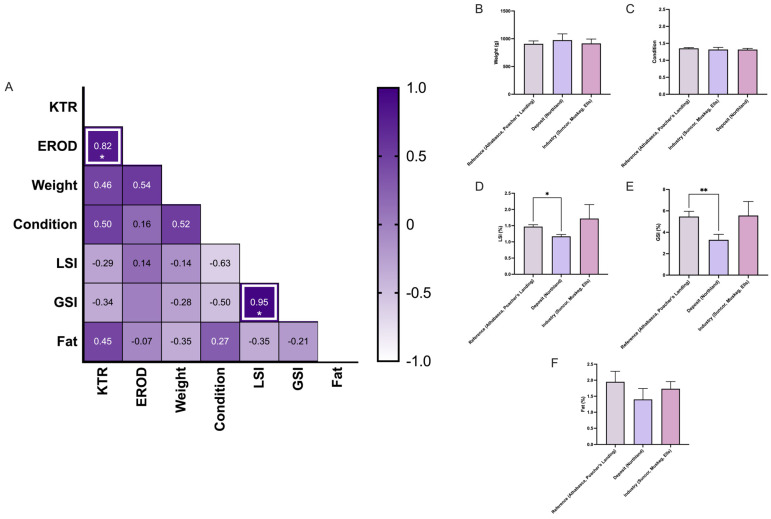
Profiling the white sucker (*Catostomus commersonii*) (**A**) Pearson’s correlation matrix analysis between ethoxyresorufin-O-deethylase (EROD) activity, kynurenine–tryptophan ratio (KTR), weight, condition, hepatic somatic index (HSI), gonadosomatic index (GSI), and fat. The heatmap color scale represents correlation coefficients (r), ranging from −1 (light purple) to +1 (dark purple). (**B**–**F**) Bar graphs of biometric indices: (**B**) weight, (**C**) condition, (**D**) Hepatic somatic index, (**E**) Gonado Somatic Index, and (**F**) fat content, measured in pooled males and females (~10 fish total) across six collection sites. Sites were grouped according to reference (Athabasca, Poacher’s Landing), oil sands deposits and sewage (Northland), and oil sands deposit and industry activity (Suncor, Muskeg, Ells). The data are presented as mean ± SEM. Statistical significance is denoted by asterisks: * *p* < 0.05, ** *p* < 0.01.

**Figure 9:**
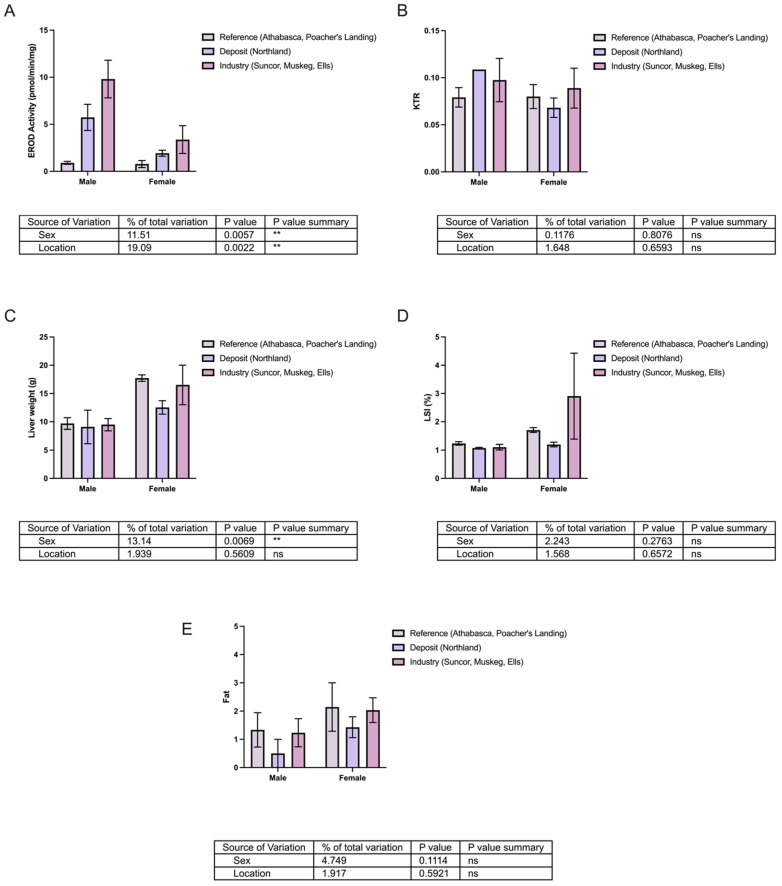
Profiling the white sucker (*Catostomus commersonii*) by sex and location. Two-way ANOVA was used to evaluate the effects of sex and location on (**A**) ethoxyresorufin-O-deethylase (EROD) activity, (**B**) kynurenine–tryptophan ratio (KTR), (**C**) liver weight, (**D**) hepatic somatic index (HSI), and (**E**) fat content. Bars represent mean ± SEM for males and females, with three bars per sex corresponding to reference sites (Athabasca, Poacher’s Landing), oil sands deposits and sewage (Northland), and oil sands deposit and industry activity (Suncor, Muskeg, Ells). Statistical significance relative to the reference group is denoted by asterisks ** *p* < 0.01.

In white suckers, there was a significant decrease in GSI at deposit sites (Figure 8), suggesting that reproductive stress in white sucker is linked to environmental conditions at the deposit sites rather than being solely driven by anthropogenic activity associated with the oil sands industry. Similarly to what was observed in longnose suckers, there was no significant correlation between GSI and KTR in white suckers (Figure 9); however, these findings are limited by the small and uneven sample sizes between sites.

## 4. Conclusions

This study provides preliminary evidence that the KTR may serve as a promising biomarker for assessing PAC exposure in aquatic environments. The observed associations between KTR and EROD activity, a well-established marker of CYP1A enzyme induction and PAC exposure, suggest that KTR has potential to reflect metabolic disruptions in response to environmental stressors linked to both natural bitumen deposits and industrial activity in the AOSR. This represents the second study to demonstrate that KTR assessments can be performed in fish, with results indicating species-specific patterns of response. Given the mandate of ECCC to advance and refine tools for environmental regulation and monitoring, further detailed studies will be important for determining whether KTR can ultimately serve as a species-specific marker of environmental and organismal health.

Future work should aim to expand sample sizes and further validate KTR across diverse environmental conditions to establish its utility as a reliable biomarker. The location-dependent nature of KTR observed in longnose suckers reinforces the need for broader temporal and spatial studies to assess the variability of KTR responses across different ecosystems, and under varying levels of industrial activity and natural deposits. While EROD activity remains a key marker for ecological assessments, KTR may provide complementary insights into energy homeostasis and downstream biological responses to xenobiotics. Future studies should assess whether KTR provides independent value or functions best in tandem with established biomarkers such as EROD. In addition, further mechanistic work is needed to examine how KTR reflects long-term metabolic impacts and reproductive health in aquatic species exposed to oil sands-related contaminants. Finally, expanding these assessments to other fish species within the region will be critical for determining the generalizability and ecological relevance of KTR as a biomarker.

Ongoing work in our group is focused on exploring the use of KTR in non-lethally collected tissues, such as mucus or scales, to enhance its application in environmental monitoring without harming fish populations. These efforts will help solidify KTR as a practical and non-invasive biomarker for assessing environmental contamination and fish health in the AOSR and beyond. Collectively, these data suggest that KTR can be used as an indicator for environmental contamination and fish health, which can further provide insights into the consequences of industrial activity on aquatic species.

## Data Availability

The original contributions presented in this study are included in the article/Supplementary Material. Further inquiries can be directed to the corresponding author.

## Supplementary Materials

The following supporting information can be downloaded at: https://www.mdpi.com/article/10.3390/toxics13100862/s1, Table S1: Sample numbers of white sucker (*Catostomus commersonii*) and longnose sucker (*Catostomus catostomus*) used in analyses, by site and sex.

## Abbreviations

The following abbreviations are used in this manuscript:

KTR	Kynurenine:Tryptophan Ratio
AOSR	Alberta Oil Sands Region
EROD	Ethoxyresorufin-O-deethylase
HSI	Hepatic somatic index
GSI	Gonadosomatic index
PAC	Polycyclic aromatic compound
ECCC	Environment and Climate Change Canada
TRP	Tryptophan
KYN	Kynurenine
SC	Sympatho-chromaffin
HPI	Hypothalamic-pituitary-interrenal
AhR	Aryl hydrocarbon receptor
