# Supplementary material for: Assessing the Kynurenine–Tryptophan Ratio (KTR) and CYP1 Activity in Longnose (Catostomus catostomus) and White Suckers (Catostomus commersonii) Exposed to Petroleum-Derived Contaminants from the Alberta Oil Sands Region
Source: Toxics. 2025 Oct 11;13(10):862. doi: 10.3390/toxics13100862 (PMC12567817; doi:10.3390/toxics13100862)
Supplement: Supplementary file 1 [file toxics-13-00862-s001.zip › toxics-3830195-supplementary.pdf]

Article

Assessing the Kynurenine–Tryptophan Ratio (KTR) and CYP1 Activity in Longnose (*Catostomus catostomus*) and White Suckers (*Catostomus commersonii*) Exposed to Petroleum-Derived Contaminants from the Alberta Oil Sands Region

Laiba Jamshed [1], Amrita Debnath [1], Amica Marie-Lucas [2], Thane Tomy [2], Gregg T. Tomy [2], Tim J. Arciszewski [3], Mark E. McMaster [4] and Alison C. Holloway [1,*]

[1] Department of Obstetrics and Gynecology, McMaster University, Hamilton, ON L8S 4L8, Canada; jamshel@mcmaster.ca (L.J.)
[2] Centre for Oil and Gas Research and Development, University of Manitoba, Winnipeg, MB R3T 2N2, Canada
[3] Alberta Environment and Parks, Calgary, AB T2E 7L7, Canada
[4] Environment and Climate Change Canada, Burlington, ON L7S 1A1, Canada; mark.mcmaster@ec.gc.ca
* Correspondence: hollow@mcmaster.ca

Supplemental:

Table S1. Sample numbers of white sucker (*Catostomus commersonii*) and longnose sucker (*Catostomus catostomus*) used in analyses, by site and sex.

Species	Site	Male (n)	Female (n)	Total (n)
Longnose sucker (*Catostomus catostomus*)	Athabasca	22	20	42
	Poacher's Landing	21	19	40
	Northland	20	20	40
	Suncor	15	23	38
	Muskeg	15	21	36
White sucker (*Catostomus commersonii*)	Athabasca	3	7	10
	Poacher's Landing	6	2	8
	Northland	2	7	9
	Suncor	4	5	9
	Muskeg	5	5	10
	Ells	2	8	10
